# Lipofection mediated transfection fails for sea urchin coelomocytes

**DOI:** 10.1371/journal.pone.0267911

**Published:** 2022-05-06

**Authors:** Megan A. Barela Hudgell, L. Courtney Smith

**Affiliations:** Department of Biological Sciences, George Washington University, Washington, DC, United States of America; Laboratoire Arago, FRANCE

## Abstract

Molecular cloning, gene manipulation, gene expression, protein function, and gene regulation all depend on the introduction of nucleic acids into target cells. Multiple methods have been developed to facilitate such delivery including instrument based microinjection and electroporation, biological methods such as transduction, and chemical methods such as calcium phosphate precipitation, cationic polymers, and lipid based transfection, also known as lipofection. Here we report attempts to lipofect sea urchin coelomocytes using DOTAP lipofection reagent packaged with a range of molecules including fluorochromes, in addition to expression constructs, amplicons, and RNA encoding GFP. DOTAP has low cytotoxicity for coelomocytes, however, lipofection of a variety of molecules fails to produce any signature of success based on results from fluorescence microscopy and flow cytometry. While these results are negative, it is important to report failed attempts so that others conducting similar research do not repeat these approaches. Failure may be the outcome of elevated ionic strength of the coelomocyte culture medium, uptake and degradation of lipoplexes in the endosomal-lysosomal system, failure of the nucleic acids to escape the endosomal vesicles and enter the cytoplasm, and difficulties in lipofecting primary cultures of phagocytic cells. We encourage others to build on this report by using our information to optimize lipofection with a range of other approaches to work towards establishing a successful method of transfecting adult cells from marine invertebrates.

## Introduction

Nucleic acid insertion into a variety of cell types has been important since the 1980s [1] and the outcomes are often beneficial for advancing basic scientific research, the pharmaceutical industry, and medical uses. Applications can include the production of mRNAs, proteins, and biopharmaceutical products for analysis or medical use including vaccines, as well as understanding or improving approaches for molecular cloning, gene manipulation, gene expression, gene regulation, and protein function [1–11]. For example, *Escherichia coli* and *Saccharomyces cerevisiae* are key organisms used in the production of insulin for treatment of diabetes (reviewed in [1]) through the introduction of the human insulin gene into these microbes [12, 13]. The process of transfecting DNA into bacterial cells was first reported by Griffith [14] when nonvirulent *Pneumococci* became virulent when injected into mice with heat killed virulent *Pneumococci*. This was later verified *in vitro* by Dawson and Sia [15, 16]. The principle of bacterial transformation was further characterized and methods were described by Avery et al. [17] followed by the development of chemical transformation for *E*. *coli* [18–22]. The transformation of DNA into *S*. *cerevisiae* was established in 1978 with the incorporation of foreign DNA into the yeast genome [23, 24]. While the transformation of foreign DNA into bacteria or yeast was groundbreaking, there were limits to its use in protein research because of post-transcriptional modifications to proteins that would normally occur in eukaryotic cells that was absent or different in bacterial or fungal cells [25, 26]. This difficulty led to establishing a number of alternative approaches to transform vertebrate and invertebrate cells.

One method for introducing exogenous nucleic acids into cells is through the use of viral-vector based methods. Viruses used in this method are manipulated by removing some viral genes encoding pathogenicity proteins while maintaining the structural genes, the regulatory regions, the genes necessary for viral replication including packing the nucleic acids into viral particles [8]. This method has excellent transfection efficiency but requires knowledge of viruses that infect the target cells or can be manipulated to do so. Common vectors used are murine leukemia virus, human immunodeficiency virus, human T lymphotrophic virus, adenovirus, adeno-associated virus, and herpes simplex virus [8].

The use of instruments is another common method to accomplish transfection into cells. All approaches have the goal of driving exogenous nucleic acids into the cell cytoplasm using physical methods that disrupt or penetrate the plasma membrane. The commonality among these methods is that there is no chemical or biological component with which the nucleic acids are associated for delivery into the cell. Instead, holes in the cell membrane facilitate the introduction of the nucleic acids into the cells, whether through a puncture using a needle as in microinjection [6, 27], or by creating pores in the membrane by electroporation through which molecules enter cells from the extracellular media [28]. Microinjection of DNA into eukaryotic cells is a popular method for introducing genes of interest into eggs and embryos to evaluate gene expression and regulation, to generate transgenic animals, or to identify intracellular signaling pathways that are active through development [27]. Most of these methods are done in mouse embryos [9], *Xenopus* embryos [10], and sea urchin embryos [29–31]. Electroporation exposes cells to a pulse of high-intensity electric field to permeabilize the membrane and introduce nucleic acids into the cells through these holes (reviewed in [28]). This method is highly efficient and can be done on a large number of cells simultaneously, unlike microinjections, to transfect large and/or small molecules such as bacterial artificial chromosomes or antibodies that do not otherwise transfect by chemical methods [28]. A solution of optimal osmolarity is necessary for electroporation and cells that require high saline solutions cannot generally be electroporated [32]. Laserfection or opto-injection are methods that function similarly to electroporation but use a laser light to permeabilize the cell membranes and create pores that allow the nucleic acid and other molecules to enter the cells from the media (reviewed in [33]). Bombardment is another means to permeabilize a cell membrane by shooting micro-particles, such as tungsten or gold, at cells at high velocity that puncture holes through which nucleic acids and other molecules can enter. This is a method that has become popular in plant transfection because it solves the problem of the plant cell wall (reviewed in [11]).

Reagent or chemical based transfection methods are all dependent on the formation of complexes between the DNA and the transfection reagent that are up taken into the target cells through endocytosis, fusion with the plasma membrane, or by osmotic shock. Some of the first experiments of chemical based transfection into eukaryotic cells involved calcium phosphate precipitation, a method that results in DNA/calcium phosphate complexes that precipitate spontaneously and are subsequently taken into target cells [4, 5, 34]. Cationic polymers or polycations (polymeric nanoparticles; PNPs) also result in complexes of anionic nucleic acids and cationic polymers to produce polyplexes. Uptake of these complexes is dependent on endocytosis or in other cases, such as diethylaminoethyl (DEAE)-dextran, can enter cells upon osmotic shock [35–39]. Activated dendrimers are similar to linear polymers but are highly branched and often spherical. They interact with DNA via charge, bind to cell membranes, and are transported into cells by non-specific endocytosis [40–42]. Magnetic beads are also used for transfection in a process known as magnetofection. Iron oxide particles complexed with nucleic acids are forced into the cells with a strong magnetic field resulting in close contact and subsequent endocytosis [43, 44].

Lipid based transfection, also known as lipofection, is another form of chemical based transfection. The potential for lipid based transfection was first deduced when lipids injected into mice not only ended up in the liver, but were found phagocytosed by Kupffer cells followed by fusion with primary lysosomes or inclusion in secondary liposomes [45]. Felgner et al. [46] went on to show that lipids, specifically *N*-[1-(2,3-dioleyloxy)propyl]-*N*,*N*,*N*-trimethylammonium chloride (DOTMA), interact spontaneously with DNA to form complexes called lipoplexes that fuse with or are endocytosed by cells or tissues in culture and thereby deliver DNA into cells. Lipoplexes are multilamellar structures that self assemble with nucleic acids and transform from liposomes into cationic lipid bilayer membranes alternating with layers of DNA [47, 48]. However, variations in lipoplex structure have been reported for different types of lipids and how they interact with DNA that can vary with the level of charge neutralization between the DNA and the cationic lipid in which the counter ions associated with both the DNA and the lipid are released into the solution during lipoplex formation [49–51]. There are a number of liposome reagents that are commercially available, which fall into three categories: cationic lipids such as DOTMA, *N*-(1-[2,3-dioleoyloxy]propyl)-*N*,*N*,*N*-trimethylammonium methyl-sulfate (DOTAP), and dioctadecylamidoglycylspermine (DOGS), neutral lipids such as 1,2-dioleoyl-*sn*-glycero-3-phosphatidylethanolamine (DOPE), 1,2-dioleoyl-*sn*-glycero-3-phosphatidylcholine (DOPC), and anionic lipids such as phosphatidic acid and phosphatidylglycerol (reviewed in [51]). Lipofection requires the formation of lipoplexes followed by incubation with target cells when the lipoplexes may fuse with the plasma membrane [46, 51] or are endocytosed by the target cells (reviewed in [47]). Lipofection reagents have been used in a number of ways to transfect nucleic acids into eukaryotic cells, however, these methods have been limited to mammalian and insect cell lines, of which both have extensive protocols for maintaining long term cell cultures in the lab [52–54]. A number of lipofection reagents are known to be cytotoxic during incubation with cells over an extended period of time, which limits their use to three hours [51]. DOTAP is not cytotoxic to mammalian cells when used below a concentration of 150 μg/mL (Roche Diagnostics). Whether this holds true for other cell types is currently unknown.

The overall goal of this work is to establish a usable lipofection protocol to incorporate nucleic acids into adult sea urchin coelomocytes for the purposes of analyzing gene expression and gene regulatory networks with the ultimate goal of understanding coelomocyte functions. To date most investigations of gene regulatory networks have focused on development of embryonic and larval sea urchins and are based on the success of microinjecting sea urchin eggs and embryos with nucleic acids (reviewed in [2]). Hence, gene regulation in adult sea urchin cells is generally unexplored. We report here on our attempts to use the DOTAP lipofection reagent to incorporate a range of molecules into sea urchin coelomocytes. Results show that DOTAP liposomes have low cytotoxicity on coelomocytes, however no signatures of successful lipofection are identified for fluorescent molecules, expression constructs containing either known or suspected *cis* regulatory regions to drive GFP expression, amplicons of the functional regions of the expression constructs, or mRNAs encoding GFP. Although our attempts to establish a protocol for lipofection into sea urchin coelomocytes fail, we report these methods and provide several possible points at which the approach may have failed so that others might use the results to avoid repeating these approaches and perhaps to advance the research with a different and viable transfection method.

## Methods

### GFP expression constructs for lipofection

The pONY_HE_GFP-X construct was provided by Drs. Jonathan Rast and Katherine Buckley (University of Toronto), which was a modified pBluescript KS(+) plasmid (GenBank accession number X52327.1) with two *Sfi*I cloning sites flanking the insert region. pONY_HE_GFP-X was digested with *Sfi*I (NEB) to release the *SpHE* insert that was separated from the pONY_X_GFP-X construct by gel electrophoresis. pONY_X_GFP-X was cut from the gel, cleaned using the QIAEX Gel Extraction Kit (Qiagen), and re-ligated with T4 DNA ligase (NEB) to generate the empty vector.

Regions of known or suspected *cis* regulatory elements for genes of interest were identified using GenePalette (http://www.genepalette.org/), a universal software tool for genome sequence visualization and analysis [55], and amplified with primers that included 5′ terminal *Sfi*I restriction sites (S1 Table). Bacterial artificial chromosome (BAC) clone R3-3033E12 (GenBank accession number BK007096) contains a tightly linked family of *SpTransformer* (*SpTrf*) genes [56–58], and a region of 3 kb located 5′ of the *SpTrf-E2* gene was amplified from BAC R3-3033E12 by PCR (S1 Table). The PCR mix included 0.5 U PrimeSTAR GXL high fidelity DNA polymerase (Takara), 1X PrimeSTAR GXL buffer, 200 μM of each dNTP, 0.2 μM of each primer (S1 Table), 10 ng BAC R3-3033E12 DNA in a final volume of 20 μL. Two other regions located 5′ of the sea urchin actin (*SpCyI*) gene with known regulatory elements [7, 59] were amplified using the same PCR protocol described above using sea urchin genomic DNA that was isolated using the CTAB method according to [60, 61]. The two *SpCyI* regulatory amplicons overlapped and included a larger amplicon of 950 nucleotides (nt) (*SpCyI-950*) and a smaller amplicon of 300 nt (*SpCyI-300*) (S1 Table). The *Sfi*I sites on the *SpCyI* and *SpTrf-E2* regulatory amplicons were opened with *Sfi*I followed by extraction with phenol/chloroform (Fisher Scientific) and passage through a G50 Sephadex spin column (Sigma). Amplicons were ligated into the *Sfi*I site of linearized pONY_X_GFP-X using T4 DNA ligase (NEB) at a 3:1 molar ratio of insert to construct/vector. The ligation mixture was used to transform TOP10 cells (Invitrogen) via heat shock and grown over night at 37°C on Luria Bertani (LB) agar plates with 100 μg/mL ampicillin (Sigma). Inserts were verified initially by size after linearizing the constructs with *Not*1 (NEB) and by sequencing the ligation sites (GeneWiz).

### Preparation of amplicons for lipofection

Amplicons used for lipofection were generated from the pONY_CyI-300_GFP-X and pONY_CyI-950_GFP-X constructs using M13 primers (S1 Table) and the PrimeSTAR GLX DNA polymerase. The amplicons contained both of the *SpCyI* regulatory region and the GFP coding region. The amplicons were isolated by gel electrophoresis followed by gel cleanup with QIAEX Gel Extraction Kit (Qiagen).

### Run-off mRNA preparation

Constructs of the pBluescript II KS+ vector containing the coding regions for either GFP or mCherry were linearized with *Sal*I (NEB) at the 3′ end or *Spe*I (NEB) at the 5′ end of the coding regions. T7 RNA polymerase (ThermoScientific) was used to generate sense strand run-off mRNA from the construct linearized at the 3′ end based on the manufacture’s protocol. T3 RNA polymerase (ThermoScientific) was used to generate run-off anti-sense mRNA from the construct linearized at the 5′ end. The mRNAs were capped with the ribo m^7^G cap mix (Invitrogen). mRNA size was verified by gel electrophoresis.

### DOTAP lipoplex formation

A variety of different molecules were packaged into the DOTAP liposomal transfection reagent (ver. 14) according to the manufacturer’s protocols (Roche Diagnostics). Nucleic acids (expression constructs, amplicons, or mRNAs) were complexed at a ratio of 6 μg DOTAP (6 μL DOTAP stock reagent) per μg of nucleic acid in 30 μl of N-2-hydroxyethylpiperazine-N-ethanesulfonic acid buffered saline (HBS; 20 mM HEPES pH 7.4, 150 mM NaCl) and incubated at room temperature for 15 minutes.

### Liposome formation with fluorescent dyes

Fluorescein isothiocyanate (FITC; Invitrogen) or rhodamine B isothiocyanate (RITC; Sigma-Aldrich) were incubated with DOTAP according to the manufacturer’s protocol using three concentrations of each fluorochrome (0.3 mg/mL, 0.03 mg/mL, or 0.003 mg/mL) to form liposomes containing each fluorochrome.

### Sea urchin care

Sea urchins, *Strongylocentrotus purpuratus*, were collected from the near-shore Pacific Ocean of Southern California, and purchased from Marinus Scientific (Long Beach, CA) or the Southern California Sea Urchin Company (Corona del Mar, CA). Sea urchins were housed for at least two years in 125 gallon marine aquaria and fed once weekly on re-hydrated kelp (Wel-Pac Dashi Kombu) and maintained as described [62].

Sea urchins do not fall under the institutional rules for ethical animal care at George Washington University because they are not vertebrates or cephalopods. No animals were sacrificed or died during the course of this study.

### Coelomocyte collection

Whole coelomic fluid (wCF; ~200–300 μL) was withdrawn from sea urchins using a 25 gauge needle attached to a chilled 1 mL syringe pre-loaded with an equal volume of ice-cold calcium- and magnesium-free seawater with 70 mM ethylenediaminetetraacetic acid (EDTA) and 20 mM HEPES (pH 7.4) (CMFSW-EH; 460mM NaCl, 10.73 mM KCl, 7.04 mM Na_2_SO_4_, 2.38 mM NaHCO_3_) [63, 64]. The wCF was adjusted to a final volume of 1 mL with additional ice-cold CMFSW-EH and expelled into a 2 mL tube on ice. Coelomocytes were counted with a TC20 automatic cell counter (BioRad) according to Chou et al. [69] and the cell concentration was adjusted depending on the experimental set.

### Lipofection of coelomocytes for microscopy

Shandon superfrost plus positively charged microscope slides (ThermoScientific) were assembled into three-chimney centrifugation holders (Eppendorf) and chilled to 4°C. Once chilled, each chimney was loaded with 200 μL of cold CMFSW-EH, into which 3 X 10^4^ cells were added, which was the optimal for an even distribution of cells without overlaps. The slide holder assemblies were centrifuged in a swinging bucket rotor (Eppendorf A-4-62) at 620 x *g* for 7 minutes at 4°C to spin the cells onto the slides and left at 4°C for an additional 5 minutes to allow cells to spread [65]. The slide holder assemblies were moved to a water-chilled cold plate that was covered with a damp paper towel to improve temperature transfer and warmed to 14°C for 5 minutes. The temperature of the cold plate was maintained by a connection to a NESLAB RTE-211 circulating chiller (Cole-Parmer). The fluid in each chimney was carefully aspirated with a glass pipette and replaced with 200 μL of coelomocyte culture medium (CCM; 0.5 M NaCl, 5 mM MgCl_2_, 1 mM EGTA, 20 mM HEPES pH 7.4) [64–66] and incubated for 5 minutes at 14°C. The CCM was replaced with 200 μL of fresh cold CCM and incubated for an additional 30 minutes at 14°C. The CCM was replaced with 200 μL of ice-chilled CCM with DOTAP liposomes or lipoplexes containing either FITC, RITC, DNA, or mRNA and incubated for 30 minutes to 6 hours at 14°C. After incubation, the CCM-liposome/lipoplex media was replaced with 200 μL of fresh chilled CCM followed by three washes of chilled CCM. The CCM was carefully aspirated after the final wash, and the slides were removed from the assemblies. Wet preparations of live coelomocytes were imaged using Zeiss Axioskop fluorescence photo-microscope (Zeiss, Oberkochen, Germany) with an attached Infinity 3 color digital camera and digital color imaging system (Lumenera).

### Lipofection of coelomocytes for flow cytometry

Eppendorf tubes (2 mL) containing 1.5 X 10^5^ coelomocytes in 1 mL of CMFSW-EH (the optimal number of cells for this analysis) were maintained at 14°C in a chiller water bath (NESLAB RTE-211, Cole-Parmer) for the duration of the experiments. DOTAP lipoplexes [5–30 μL) with the GFP expression constructs and control liposomes without DNA were chilled on ice for 5 minutes before adding to the coelomocytes. Cells and DOTAP liposomes or lipoplexes were mixed immediately by inverting the tubes slowly twice, and returning to 14°C. The cells plus DOTAP liposomes/lipoplexes were inverted every 30 minutes over the span of 3 hours to ensure that the lipoplexes and the cells remained in suspension. Sub-samples were taken every hour to evaluate cell viability. After the initial 3 hours, some samples were incubated for 12–36 hours. For some analyses, the buffer was adjusted to 460 mM NaCl to compensate for adding the DOTAP liposomes/lipoplexes in lower ionic strength to the cells.

Coelomocytes incubated with liposomes/lipoplexes were evaluated by flow cytometry as described [63, 67] using an Accuri C6 Flow Cytometer (BD Biosciences). Coelomocytes were incubated with propidium iodide (PI; 1 μg/mL) on ice for 5 minutes followed by flow cytometry with initial gate parameters set to complexity vs. size (side scatter—area [SSC-A] vs. forward scatter—area [FSC-A]). Additional parameters were established to gate out cells positive for PI, cell doublets (SSC-A vs. side scatter—height [SSC-H]), and debris prior to further analysis. All remaining events were deemed to be live coelomocytes and were analyzed and gated for green fluorescence using FlowJo software (https://www.flowjo.com).

### Statistical analysis

Two-tailed, unequal variance, unpaired *t*-tests, and one-way ANOVA were carried out in Excel (Microsoft) and used to determine significant differences among challenged and control groups, which were standardized based on coelomocytes per sample. Quartile and τ tests were used to identify outliers that would indicate a significant change in viability upon treatment with DOTAP compared to untreated controls. Binomial test of significance was used to determine the significance of the averaged proportions among challenged and control groups. The null and alternative hypothesis were H_0_: p1 = p2 and H_1_: p1 ≠ p2. Significance was set at *p* ≤ 0.05 for all ANOVA, *t*-tests, and binomial test of significance.

## Results

### DOTAP is not toxic to coelomocytes

**Fig 1:**
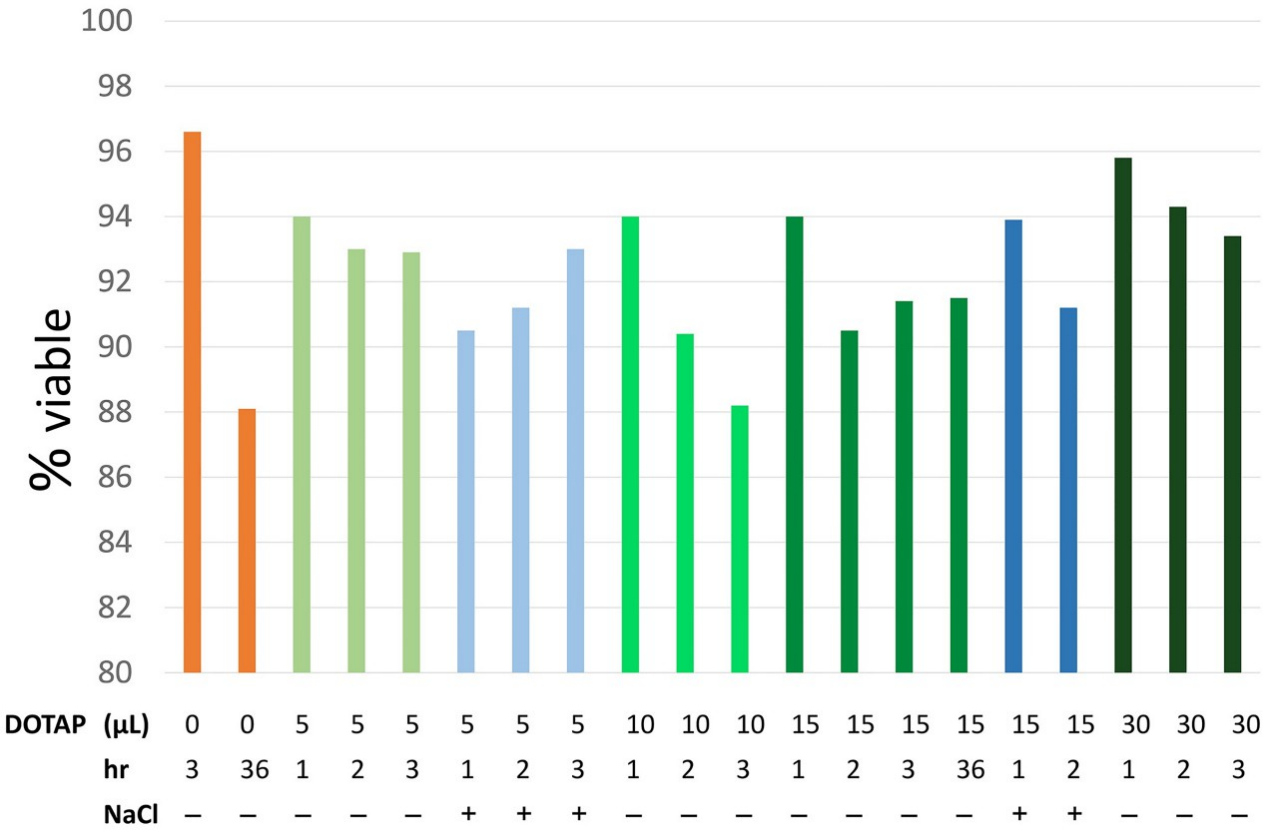
The percentage of viable coelomocytes over 36 hours does not change when incubated with DOTAP. Cell viability was gauged using propidium iodide exclusion after incubation with DOTAP. Coelomocyte suspensions (1.5 X 10^5^) were treated with 0 to 30 μL of DOTAP and tested for viability at 1, 2, 3 and 36 hours (hr). The salinity of the medium was adjusted for some samples by adding NaCl to compensate for the addition of DOTAP in HBS.

Little is known about the effects of lipofection reagents on sea urchin coelomocytes or cells from other marine animals. Therefore, we first evaluated whether DOTAP was cytotoxic to coelomocytes from the purple sea urchin, *Strongylocentrotus purpuratus*. DOTAP was selected for this study because of its reported low levels of cytotoxicity. Cell samples were exposed to varying amounts of DOTAP liposomes over a three-hour period plus a final analysis at 36 hours and evaluated by PI staining and flow cytometry to determine the level of toxicity for coelomocytes. Cells that received 5 μL, 10 μL, 15 μL or 30 μL of DOTAP liposomes (which ranged from half to twice the volume recommended by the manufacture relative to the number of cells) showed a decrease in cell viability after 1 to 36 hours of incubation (Fig 1, Table 1). Coelomocytes incubated with 5 to 30 μL of DOTAP for 1 to 36 hours showed viability within the range of that for control cells that were incubated in the absence of DOTAP (Fig 1; all green bars). The lowest cell viability was observed with 10 μL of added DOTAP, which was the only sample that showed a continuous decrease in cell viability over 3 hours (Fig 1; bright green bars). Coelomocytes that were incubated with 15 μL of DOTAP (the manufacturer recommended volume) were extended to 36 hours to test their viability over this longer time period. Control cells without DOTAP showed 88% viability over 36 hours, whereas coelomocytes with DOTAP showed 91.5% viability indicating that DOTAP did not impact the viability of these cells in short term culture. Outlier tests were used to demonstrate that none of the experimental samples were statistical outliers and all were within the range of viability for the controls in the absence of DOTAP. Although incubation with DOTAP resulted in an initial decrease in cell viability after an hour, the decrease was similar to non-treated coelomocytes. Based on these results, the toxicity of DOTAP on coelomocytes was deemed to be minimal and could be used for further evaluation of lipofection.

**Table 1:** Percent cell viability after treatment with DOTAP.

DOTAP added per treatment	Gated cells counted^1^	Viable (%)
0 μL		
3 hr	5000	96.6
36 hr	5000	88.1
Unstained^2^	5000	99.8^3^
5 μL		
1 hr	5000	90.5
2 hr	5000	91.2
3 hr	1970	93.0
1 hr + NaCl	5000	94.0
2 hr + NaCl	5000	93.0
3 hr + NaCl	2213	92.9
10 μL		
1 hr	5000	94.0
2 hr	5000	90.4
3 hr	2240	88.2
15 μL		
1 hr	5000	94.0
2 hr	5000	90.5
3 hr	2006	91.4
36 hr^4^	5000	91.5
1 hr + NaCl	5000	93.9
2 hr + NaCl	5000	91.2
3 hr + NaCl	No data^5^	No data^5^
30 μL		
1 hr	5000	95.8
2 hr	5000	94.3
3 hr	5262	93.4

^1^The numbers of coelomocytes within the gate for viable cells that exclude PI were counted and are indicated for each sample.

^2^The control sample was not incubated with PI.

^3^The percentage of cells in the gate established for cells that exclude PI is used to evaluate mostly live cells when PI staining and exclusion is not used.

^4^This sample was evaluated at 36 hours based on the manufacturer’s recommendation of liposome volume and ratio of liposomes per cell.

^5^Not enough coelomocytes were recorded for these samples to reach 5000 events.

### Salinity adjustments to the DOTAP buffer are not required

Coelomocytes are maintained in short term cultures in high salinity CCM because coelomic fluid salinity is equivalent to seawater (0.46 M NaCl) [64, 65, 68–71], whereas the DOTAP liposome mixture is used at mammalian salinity (0.15 M NaCl). Although the volume of liposomes added to the coelomocytes was low (25 μL to 150 μL of DOTAP added to 1 ml of cells), the salinity of the solution may have impacted the cells. Therefore, we tested whether the decrease in salinity by adding the DOTAP liposomes to the cells might induce cellular clotting reactions or be a source of reduced viability. The salinity of the DOTAP liposomes was adjusted to 0.46 M NaCl before adding to the coelomocytes. Two sets of samples with equal numbers of coelomocytes in 1 mL of CMFSW were used, one receiving 25 μL (Fig 1, light blue bars) and another 75 μL of the liposome mixture (Fig 1, dark blue bars). These samples were selected based on the manufacture’s recommended minimum amount (5 μg) and standard amount (15 μg) of liposome added to cells per mL. There was no distinguishable difference in cell viability in samples that were adjusted for salinity (Fig 1; all blue bars) compared to the non-adjusted samples (Fig 1; all green bars). Therefore, the decreased salinity of the DOTAP liposomes did not have deleterious effects on coelomocyte viability and adjusting the salinity of the DOTAP liposomes prior to adding to the cells was not necessary.

### FITC and RITC are incorporated into liposomes but may not be transferred into coelomocytes by lipofection

FITC and RITC were used initially to test DOTAP lipofection into coelomocytes either through fusion with the plasma membrane or, more likely, through endocytosis of the liposomes. FITC and RITC were selected to visualize their incorporation into liposomes and whether lipofected coelomocytes became fluorescent. Fluorescence was observed in the DOTAP liposomes indicating that the FITC and RITC had been incorporated into the liposome lumens. Coelomocytes on slides were incubated with liposomes for 30 minutes prior to evaluation by fluorescence microscopy. Liposomes containing either FITC or RITC appeared to be associated with the plasma membrane of coelomocytes, however, it could not be determined definitively whether liposomes were in contact with cell surfaces or whether the fluorescent dyes were incorporated into the coelomocyte cytoplasm. Varying the concentration (0.3 mg/mL, 0.03 mg/mL, or 0.003 mg/mL) of the fluorescent dyes in the liposomes did not change the outcome or make it possible to visualize the fluorescent dyes in the cytoplasm of the coelomocytes. While, and liposomes appeared to be associated with the coelomocytes, direct contact and uptake by the coelomocytes and release of fluorescence into the cytoplasm could not be verified. Therefore, this approach for using fluorochromes could not be used to confirm the success or failure of lipofection.

### Transfection with expression constructs does not result in expression of fluorescent proteins

**Fig 2:**
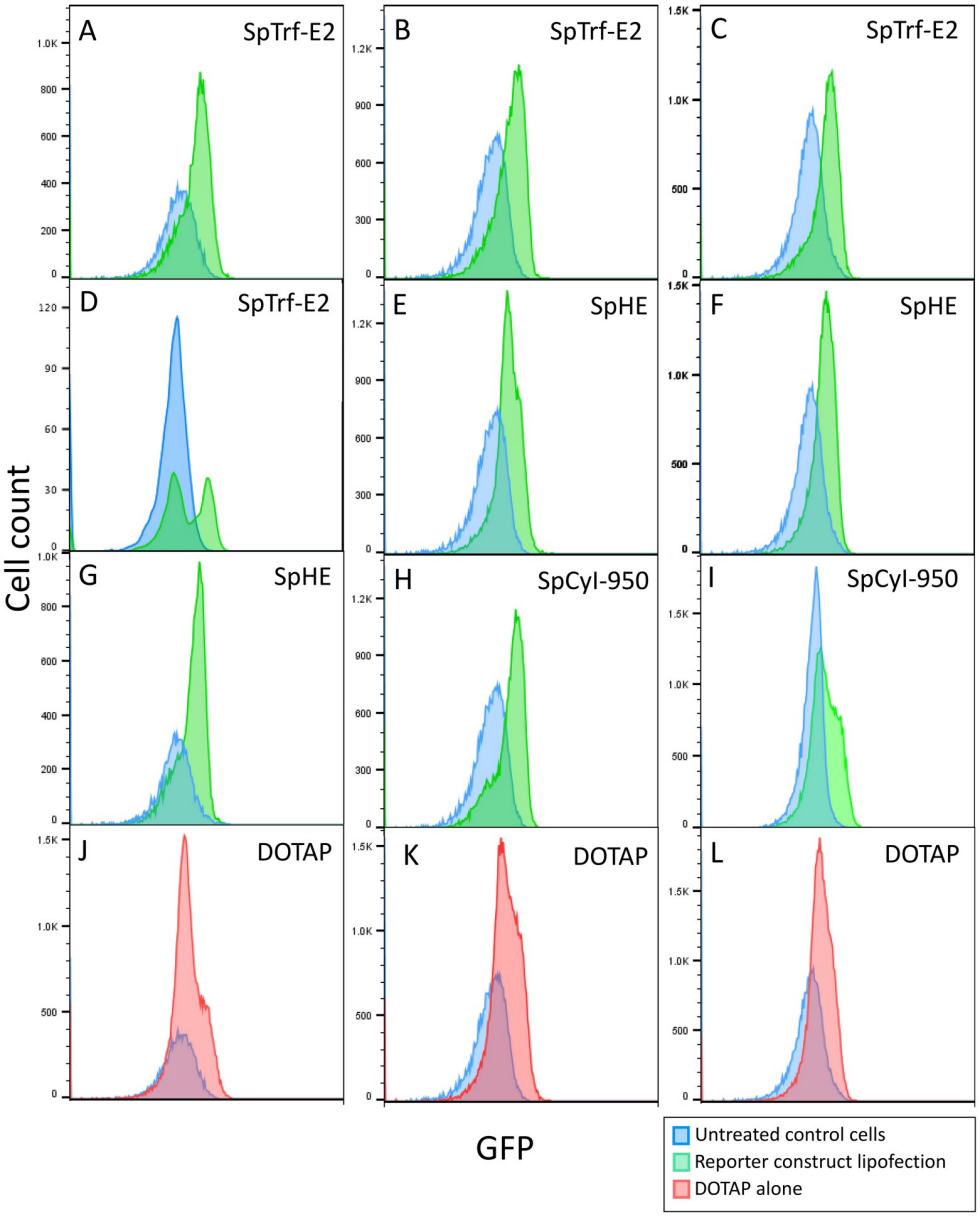
Green fluorescence in coelomocytes is increased in samples lipofected with expression constructs to drive GFP expression. Flow cytometry histograms for green fluorescence are shown for replicate samples of coelomocytes incubated with DOTAP lipoplexes with one of the GFP expression constructs (A-I; green), or with DOTAP liposomes without DNA (J-L; red), or were not treated (blue). Histograms of lipofected coelomocytes are overlaid on histograms of untreated control cells.

The analysis of lipofection of FITC and RITC with DOTAP failed to provide conclusive evidence that the liposomes were associated directly with the coelomocyte surfaces or that fluorochromes were incorporated into the cytoplasm. Consequently, lipofection of expression constructs was used as the next approach because GFP expression could be evaluated by flow cytometry. *cis* regulatory elements that could drive expression of fluorescent proteins were employed. The pONY_HE_GFP-X expression construct included a ubiquitously expressing *cis* regulatory element that controls expression of the sea urchin hatching enzyme (SpHE), which is a protease expressed early in sea urchin development that digests the egg fertilization envelope [72–74]. This *cis SpHE* regulatory element is often used as a positive control in larval sea urchin gene regulatory experiments because it drives constitutive expression in all cell types [73, 75]. The pONY_CyI-300_GFP-X and pONY_CyI-950_GFP-X expression constructs included two overlapping *cis* regulatory elements of the sea urchin actin (*SpCyI*) gene [7, 59], which is expressed in coelomocytes [76]. Lastly, a region of predicted *cis* regulatory elements for an *SpTrf* gene (encoding an SpTrf protein with an E2 type sequence) from the *SpTrf* gene family was selected and inserted into pONY_SpTrf-E2_GFP-X to drive GFP expression. The *SpTrf* family of immune genes is upregulated in coelomocytes and larval blastocoelar (immune) cells upon immune challenge [65, 76, 77] and the *SpTrf*-*E2* genes show the highest expression in adult sea urchin coelomocytes compared to other *SpTrf* genes [60, 78]. These constructs were incorporated into DOTAP lipoplexes, incubated with coelomocytes, and the cells were screened for GFP expression by flow cytometry. To enable the evaluation by flow cytometry, coelomocytes were incubated with DOTAP lipoplexes while in suspension rather than on glass slides as used for microscopy (see Methods). After incubation with 15 μL of lipoplexes or liposomes without DNA for 12–16 hours, results flow cytometry showed that all expression constructs, pONY_HE_GFP-X, pONY_SpTrf-E2_GFP-X, and pONY_CyI-950_GFP-X, had similar levels of detectible green fluorescence (Fig 2, Table 2) with no statistical variation in intensity among the groups (Table 3; first three columns). Coelomocytes that received expression constructs in lipoplexes had a significantly greater percentage of cells with higher fluorescence intensity compared to untreated control cells and cells that only received DOTAP liposomes suggesting that GFP was expressed (Fig 2A–2I, Tables 2 and 3). However, green fluorescence was also observed in 47% to 65% of the coelomocytes incubated with DOTAP liposomes without DNA, which was significantly different from untreated control cells that also showed 5% to 42% fluorescent cells (Fig 2J–2L, Tables 2 and 3). This suggested that an unknown interaction between the coelomocytes and the DOTAP liposomes and lipoplexes resulted in significant background fluorescence, and that control cells in the absence of DOTAP also showed signs of auto-fluorescence. DOTAP in the absence of cells is not fluorescent by microscopy in accordance with descriptions of the product by the manufacturer. The combination of the elevated level of background fluorescence from the liposomes plus coelomocyte auto-fluorescence resulted in inconclusive lipofection results. However, the significant differences in fluorescence for the coelomocytes that received expression constructs in lipoplexes versus those that received DOTAP liposomes (Fig 2A–2I vs. Fig 2J–2L) suggested that lipofection of GFP expression constructs may have been successful and that GFP was expressed and produced by some of the coelomocytes.

**Table 2:** The percentage of GFP^+^ coelomocytes lipofected with GFP expression constructs is variable among samples^1^.

Sea urchin	Treatment	Total coelomocytes	GFP^+^ coelomocytes	GFP^+^ cells (%)
**1**	pONY_SpTrf-E2_GFP-X	28665	21504	75
**1**	pONY_HE_GFP-X	34749	25338	73
**1**	DOTAP alone	47538	27787	58
**1**	Control	30453	3392	11
**2**	pONY_SpTrf-E2_GFP-X	23311	16961	73
**2**	pONY_HE_GFP-X	26346	17309	66
**2**	DOTAP alone	41051	19407	47
**2**	Control	14863	994	7
**3**	pONY_SpTrf-E2_GFP-X	22977	18376	80
**3**	pONY_HE_GFP-X	47876	38416	80
**3**	DOTAP alone	63458	40989	65
**3**	Control	19967	8384	42
**4**	pONY_SpTrf-E2_GFP-X	28691	20804	73
**4**	pONY_CyI-900_GFP-X	22683	15235	67
**4**	pONY_HE_GFP-X	51194	36932	72
**4**	DOTAP alone	63547	33488	53
**4**	Control	12208	922	8
**5**	pONY_SpTrf-E2_GFP-X	33256	24192	73
**5**	pONY_CyI-900_GFP-X	29687	21649	73
**5**	pONY_HE_GFP-X	33665	24721	73
**5**	DOTAP alone	48505	29811	61
**5**	Control	28189	1544	**5**
**6**	Control	37828	10785	29
**6**	pONY_CyI-900_GFP-X	40213	26719	66

^**1**^These data were acquired by flow cytometry.

**Table 3:** There are significant differences in GFP expression among cells that received lipoplexes, DOTAP liposomes, or were untreated^1^.

Sample 1^2^	Sample 2	*t*-test^3^	Anova^3^	Binomial
pONY_SpTrf-E2_GFP-X	pONY_CyI-900_GFP-X	0.083	0.044	Fail to Reject
pONY_SpTrf-E2_GFP-X	pONY_HE_GFP-X	0.469	0.465	Fail to Reject
pONY_CyI-900_GFP-X	pONY_HE_GFP-X	0.238	0.265	Fail to Reject
pONY_SpTrf-E2_GFP-X	DOTAP alone	0.003	0.0008	Reject
pONY_CyI-900_GFP-X	DOTAP alone	0.021	0.037	Reject
pONY_HE_GFP-X	DOTAP alone	0.004	0.003	Reject
pONY_SpTrf-E2_GFP-X	Untreated Control	0.0002	1.5 E-05	Reject
pONY_CyI-900_GFP-X	Untreated Control	0.0002	0.0008	Reject
pONY_HE_GFP-X	Untreated Control	0.0001	2.5 E-05	Reject
DOTAP alone	Untreated Control	0.0006	0.0004	Reject

^1^These data were acquired by flow cytometry.

^2^GFP expression (Table 2) for pairs of samples are compared to determine significant difference.

^3^*p* values; *p <* 0.05 is considered significant.

**Fig 3:**
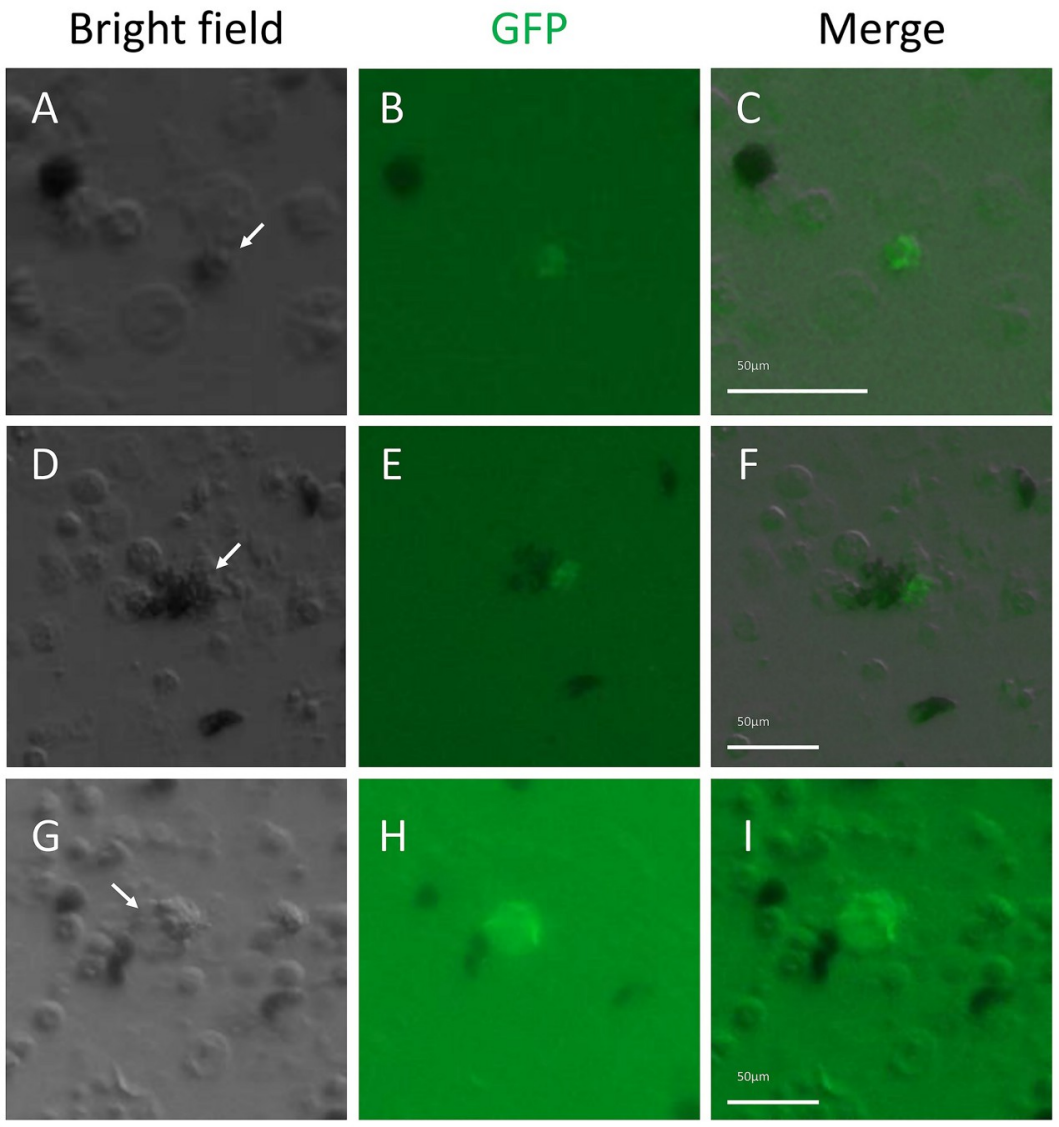
Coelomocytes lipofected with the pONY_HE_GFP-X expression construct or the amplicon show low levels of GFP expression. (A-F) A few selected cells show green fluorescence after lipofection with the pONY_HE_GFP-X expression construct. (G-I) Similarly, only a few cells show green fluorescence after lipofection with the HE_GFP-X amplicon. The white arrows in (A), (D), and (G) indicate GFP expressing cells in (B), (E), and (H). Scale bars in the merge images apply to the other panels in the same row.

To verify the flow cytometry results, coelomocytes were prepared as described in the methods for microscopy and incubated for 6 hours with lipoplexes containing either the pONY_HE_GFP-X expression construct or the corresponding amplicon of the functional regions of this construct. Control cells were treated similarly with DOTAP liposomes without DNA. Cells were evaluated for GFP expression by fluorescence microscopy. Few to no cells were observed with GFP fluorescence (Fig 3, Table 4), which contradicted results from flow cytometry showing a large portion of coelomocytes expressing GFP after receiving lipoplexes with the HE expression construct. However, flow cytometry also showed that some coelomocytes were also fluorescent after incubation with DOTAP liposomes, or the cells were auto-fluorescent. The results from microscopy indicated that there was no true GFP fluorescence by the lipofected coelomocytes. It was not determined, however, whether this outcome was due to a failure of lipofection, a failure of gene expression, or a failure to translate the mRNA into GFP. To test the function of the expression constructs to drive GFP expression, they were injected into sea urchin eggs, which were allowed to develop to the pluteus larval stage. Larval expression of GFP from pONY_SpTrf-E2_GFP-X was restricted to the larval blastocoelar cells (S1A–S1C Fig) in agreement with previous reports [77]. Larval expression of GFP from pONY_HE_GFP-X was random and consistent with non-specific expression control and the mosaic incorporation of the expression construct into the genomic DNA of a subset of embryonic cells (S1D–S1F Fig) [75, 79]. Larvae injected with the empty vector, pONY_X_GFP-X (S1G–S1I Fig), or were not injected (S1J–S1L Fig) showed background fluorescence mostly in the gut. These results indicated that the regulatory regions inserted into the expression vectors functioned as expected in larval sea urchin cells to drive GFP expression above background. Therefore, the basis for the green fluorescence of the coelomocytes detected by flow cytometry was not due to the DOTAP lipoplexes, and its origin is unknown. These results indicated that coelomocytes lipofected with the expression constructs did not express GFP.

**Table 4:** Coelomocytes do not express GFP after transfection with expression constructs or with mRNA encoding fluorescent proteins.

Lipofected nucleic acids	Number of fluorescent cells	Number of cells evaluated	Fluorescent cells (%)
DOTAP alone	0	3x10^4^ (x3)^2^	0
pONY_SpTrf-E2_GFP-X	4	3x10^4^ (x2)	0
pONY_CyI-900_GFP-X	0	3x10^4^ (x2)	0
pONY_HE_GFP-X	2	2.3x10^5^	0.001
Amplicon HE_GFP-X	3	2.3x10^5^	0.001
Linear pONY_HE_GFP-X	2	2.3x10^5^	0.001
pONY_X_GFP-X	0	2.3x10^5^	0
Amplicon X_GFP-X	0	2.3x10^5^	0
Linear pONY_X_GFP-X	0	2.3x10^5^	0
GFP anti-sense mRNA	0	3x10^4^	0
GFP sense mRNA	1	3x10^4^	0.003
mCherry anti-sense mRNA	0	3x10^4^	0
mCherry sense mRNA	4	3x10^4^	0.01

^1^These data were collected by fluorescence microscopy.

^2^Number of replicates.

### Linearized constructs do not increase the number of coelomocytes with detectible GFP fluorescence

Because of the failure to observe GFP fluorescence in coelomocytes after lipofection of expression constructs, we considered whether transcription of supercoiled DNA might have been a basis for this failure. This notion was based on the use of linearized constructs for injection into sea urchin eggs, even though linear DNA is used to promote concatenation and insertion of the DNA into the genome of embryos [80]. We hypothesized that linear DNA might be more accessible for the assembly of the transcription complex compared to a supercoiled plasmid. Therefore, all GFP expression constructs were linearized, in addition to amplicons of the GFP coding region with and without the associated promotor region, were incorporated into DOTAP lipoplexes. Cells were evaluated by fluorescence microscopy for the expression of GFP; flow cytometry was not used for this analysis based on the background levels described above. Although low fluorescence was detected in a few cells that received either the linear pONY_HE_GFP-X expression construct or the corresponding amplicon (Fig 3A–3F, Table 4), which was similar to cells receiving the supercoiled constructs (Fig 3G–3I, Table 4). These results showed that lipofection of linearized constructs or amplicons failed to induce significant GFP expression in the coelomocytes.

### Lipofection of mRNA does not produce fluorescence in coelomocytes

**Fig 4:**
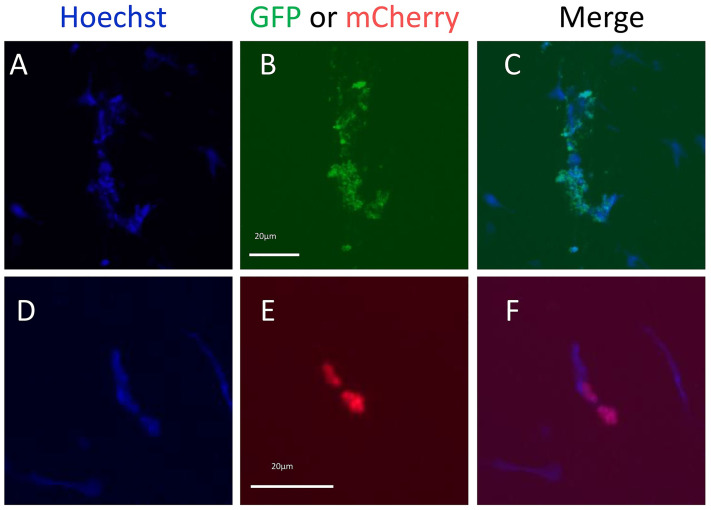
Coelomocytes lipofected with mRNA encoding GFP or mCherry result in very few examples of fluorescent cells. (A-C) Coelomocytes lipofected with GFP sense strand mRNA. (D-F) Coelomocytes lipofected with mCherry sense strand mRNA. (B) Selected coelomocytes show GFP green fluorescence. (E) Selected coelomocytes show mCherry fluorescence. The scale bar in (B) applies to panels (A) and (C). The scale bar in (E) applies to panels (D) and (F). Results for all fluorescent cell instances and controls are shown in Table 4.

To determine whether the failure to detect GFP mRNA in coelomocytes was due to a failure of lipofection or a failure to transcribe the lipofected DNA, run-off transcripts of sense and anti-sense mRNA for GFP and mCherry were generated and assembled into DOTAP lipoplexes. Coelomocytes were incubated for 6 hours with DOTAP lipoplexes containing the transcripts followed by evaluation by fluorescence microscopy. Results showed that very few coelomocytes expressed either GFP or mCherry proteins (Fig 4) and that there were few differences between cells lipofected with sense vs. anti-sense strand mRNAs for GFP (0.003% vs. 0%) or mCherry (0.01% vs. 0%; Table 4). Overall, these results indicated that by circumventing transcription, lipofection of mRNA encoding GFP or mCherry did not result in a detectible level of fluorescence in coelomocytes. Taken together these results suggested that nucleic acids were not incorporated into coelomocytes using DOTAP lipoplexes.

## Discussion

The ability to introduce nucleic acids into coelomocytes would open avenues for investigations of gene expression in adult sea urchin cells, perhaps leading eventually to a characterization of their gene regulatory networks. Gene expression in adult cells could then be compared to the regulatory networks in larval sea urchins [2] including cells that function in the larval immune system [75, 77, 81]. Understanding gene expression in adult coelomocytes could also be used to predict their functions in the echinoid immune system. This information could be applied to defining functional differences among cells of the same or different morphotypes, and could elaborate on how immune genes are regulated in coelomocytes based on the triggers that activate various immune responses. Modifications to lipofection methods for marine animals that live in elevated salinity could be applied to other invertebrates to improve the understanding of their biology. While our approaches failed to lipofect macromolecules into coelomocytes, it is important to describe our results so that others avoid repeating this approach and perhaps will use our failures to focus on finding alternative solutions for successful transfection of adult sea urchin cells.

### DOTAP does not transfect sea urchin coelomocytes

While DOTAP shows low toxicity for coelomocytes and has no requirement for minor adjustments to the salinity of the CCM after auto-assembly of the liposomes or lipoplexes in 150 mM NaCl, the results presented here indicate that DOTAP does not transfer nucleic acids or fluorochromes into the cytoplasm of coelomocytes at a level at which GFP can be observed and verified by fluorescence microscopy. It is noteworthy that a difference in sensitivity of a fluorescence microscope compared to detection by flow cytometry, which is currently considered to be more sensitive [82] as has been reported for studies of sperm, shows a disconnect between these two methods to evaluate cell fluorescence [83]. This may explain some of the differences observed between the results from microscopy and flow cytometry emphasizing that each detection method must be evaluated carefully and verified using alternative approaches.

The chemistry of the interactions and stability between cationic lipids such as DOTAP and DNA, which results in a neutralization of the charges associated with the cationic lipid and the DNA phosphate backbone in low salt solutions, has rarely been investigated in high salt media such as CCM that is required to maintain coelomocytes. Although the effect of 0.5 M NaCl on the chemistry of lipoplexes has not been evaluated directly, 1.5 M NaCl results in a partial dissociation of DOTAP and the DNA [84]. Furthermore, it is not known whether the 0.5 M NaCl in CCM and the associated ionic strength of the media disrupts, interferes, or weakens the electrostatic interactions between the lipids and the nucleic acids that i) drive the release of the counterions and water molecules associated with the lipid and the DNA and maintain the lipoplex structure, ii) whether the lipoplexes remain intact or release some or all of the DNA, and iii) whether they interact with the negatively charged cell membrane that is based on the positive charge of the DOTAP head group [85–87].

It is generally accepted that lipoplexes are endocytosed and the failure to release the expression constructs from endocytic vesicles into the cytoplasm may be related to the coelomocyte functions as immune cells. Lipofection of primary cultures of immune cells such as mammalian macrophages, dendritic cells, and other leukocytes have generally failed when commercial lipofection reagents are used [88–92]. Lipofection success for this general cell type has required significant effort for optimization using a variety of lipid mixtures or other approaches. The coelomocytes evaluated here are a highly phagocytic subtype [63, 76] and they would be expected to take up foreign materials readily, including lipoplexes and liposomes, and degrade them through the endosomal-lysosomal pathway, as has been suggested for many other cell types [47]. The high NaCl concentration in the CCM may change the electrostatic interactions in the lipoplexes and alter their sizes, which will determine whether they may be taken up by clathrin-mediated endocytosis or micropinocytosis, or whether they may enter the cell by caveolae-mediated endocytosis [93]. If the high salt concentration causes the lipoplexes to aggregate into larger particles, they may be phagocytosed by the coelomocytes that also lead to degradation in phagolysosomes. The mechanism by which lipoplexes enter a cell defines the trafficking pathway and whether they will be degraded in a lysosome, or whether the nucleic acids will be released into the cytoplasm. Regulating the size of the liposome in addition to the lipid composition may be key to lipofection success for cells from marine invertebrates (see [93] and references therein).

An essential and rate-limiting step in lipofection is the escape of the lipoplexes and from the endosomal vesicles and the release of the nucleic acids into the cytoplasm prior to degradation in the lysosome [94–97]. The escape success of DNA from the endosomal system has been estimated to be 0.01% to 1% of the amount that is endocytosed by a cell [96]. The possible lack of endosomal escape for nucleic acids in highly phagocytic immune cells, in addition to the multiple effects of the high salt media on lipoplex structure, size, and interactions with the cell surface may all be involved in why lipofection fails to produce observable GFP or mCherry fluorescence in the coelomocytes. Although other types of adult echinoid cells that are not phagocytic immune cells might be used for lipofection testing to improve survival of the lipoplexes upon uptake, coelomocytes are by far the easiest cell type to collect and maintain [63] and does not require sacrificing the sea urchin. Primary cells will be required for further efforts to solve the lipofection method because there are no echinoderm cell lines. Furthermore, if there are multiple barriers to lipofection, then the use of other transfection lipids such as different lipid mixtures that may regulate the size of the lipoplexes, or the addition of helper lipids such as DOPE or cholesterol, and vesicle lytic or escape agents should be investigated for improved results compared to those reported here [98–100].

## Conclusion

DOTAP does not result in successful lipofection of nucleic acids or fluorochromes into coelomocytes using the methods and approaches described here. Testing and optimizing lipofection for marine phagocytes in a high salt medium was beyond the scope of this study, yet these results are reported as a starting place for others to find alternative approaches to transfect adult marine invertebrate cells. We show that DOTAP is non-toxic to coelomocytes, and therefore it has potential for use in conjunction with alternative mixtures of lipids and other reagents to transfect sea urchin coelomocytes as has been reported in other model systems [51].

Careful reading of the relevant literature to understand how lipoplexes assemble [50] and are employed for transfecting cells (reviewed in [101]), indicate a number of points to consider prior to employing modifications to the method that we report. i) Differences in ionic strength of the media or buffer can dictate the shape and size of the lipoplexes, which are defined by ionic interactions between the lipids and the nucleic acids, in addition to whether the lipoplexes dissociate and release the nucleic acids [84–86]. Furthermore, the ratios of lipid to nucleic acid should result in neutralization of charges to avoid aggregation of lipoplexes [102]. Direct observations of the lipoplexes should be carried out to characterize the structure, shape, and size prior to use with cells using cryo- or standard transmission electron microscopy, light microscopy, plus indirect analyses by dynamic light scattering, among other methods as described by [87, 101, 102]. If possible, altering the ionic strength of the buffer or medium to reduce the lipoplex sizes to small enough for uptake preferentially by the caveolae system should improve transfection success [100, 101]. Targeting this pathway would avoid degradation in lysosomes that follows phagocytosis and endocytosis. ii) Because the initial interaction between lipoplexes and cells occurs at the cell surface, an understanding of the glycocalyx structure will be important [50, 101]. Different cell types from a species, cells from different organisms, and primary cultures vs. immortal cell lines are speculated to be quite different both structurally and biochemically with direct impacts on lipofection success [87]. These differences may include variations in the lipids that are present in the outer leaflet of the plasma membrane, and the oligosaccharides that are linked to glycoproteins, glycolipids, and proteoglycans that make up the glycocalyx. These examples of cell-specific variations may require the use of different mixtures of lipids including ‘helper’ or co-lipids to optimize lipofection [87] for a particular cell type. iii) The addition of reagents that enable liposomes or lipoplexes to escape from the endosomal vesicle of phagosome, or the use of endosomal escape domains can be employed with any type of macromolecule to improve lipofection success [98, 99, 103]. These agents may be particularly useful if the size of the lipoplexes cannot be manipulated by altered ionic strength so that the lipoplexes are more likely to be taken up into the endosomal or caveolae systems. In general, lipoplex sizes are sensitive to the mixture of lipids that are used, the charge ratio of the nucleic acids and the lipids, the order in which the nucleic acids and lipids are mixed, and the ionic strength of the medium [87, 104].

In light of the difficulties in using lipofection, it is worth noting that it is not the only available method of transfection; laserfection, particle bombardment, or other chemical based transfection methods such as calcium phosphate, cationic polymers, or magnetofection may be considered and evaluated as alternative approaches. Sea urchin coelomocytes are, in limited ways, optimal for this type of analysis because they are easy to obtain and established protocols are available to maintain them for days to weeks in culture [63, 64, 105]. Although the approaches and results presented here demonstrate that DOTAP lipofection fails, we provide an initial roadmap for others to work toward establishing a successful method to lipofect adult sea urchin cells and adult cells of other marine invertebrates.

## Supporting information

S1 Table Primers used to amplify the regulatory regions for the GFP expression constructs. (PDF)

S1 Fig Pluteus sea urchin larvae injected with *GFP* expression constructs show GFP expression. Eggs were injected with expression constructs according to [80] and evaluated at six days post fertilization as pluteus larvae. **A-C**. A larva injected with pONY_SpTrf-E2_GFP-X shows GFP expression in blastocoelar cells, in agreement with Ho et al. [77]. **D-F**. A larva injected with pONY_HE_GFP-X shows constitutive but mosaic GFP expression, in agreement with Solek et al. [75]. **G-I**. A larva injected with the empty vector, pONY_X_GFP-X, shows regions of auto-fluorescence, mostly in the gut. **J-L**. A non-injected larva shows regions of auto-fluorescence, mostly in the gut. Scale bars indicate 100 μm.
(PDF)
