# Supplementary material for: Lipofection mediated transfection fails for sea urchin coelomocytes
Source: PLoS One. 2022 May 6;17(5):e0267911. doi: 10.1371/journal.pone.0267911 (PMC9075664; doi:10.1371/journal.pone.0267911)
Supplement: S1 Table — (PDF) [file pone.0267911.s001.pdf]

Supporting Information MA Barela Hudgell and LC Smith

Supporting Table

Table S1. Primers used to amplify the regulatory regions for the GFP expression constructs

Primer	Sequence[1]
SpTrf-E2F	*aaa**GGCCcatgcGGCC*** AAACAATCATCATCAATTTTCTAATCA
SpTrf-E2R	*aaa**GGCCggccgGGCC*** ACYCRTCTGCATTCCAYC
CyIpony300F	*aaa**GGCCcatgcGGCC*** GGGGTGTAGAGTGCTTGATC
CyIpony950F	*aaa**GGCCcatgcGGCC*** TGGTTCAACATTTCAAGTTACTGTT
CyIponyR	*aaa**GGCCggccgGGCC*** AGCTTTGCATGCGATGTGTA

[1]Lower case *aaa* indicate added nucleotides to improve binding and digestion by the restriction enzyme. The bold font indicates the *Sfi*I restriction site (GGCCNNNNNGGCC), which has variable internal sequence. The lower case bold letters indicate the sequences chosen for use in the N region of the cleavage site. N, any base; R, C or T; Y, A or G.
