# Supplementary material for: Lipofection mediated transfection fails for sea urchin coelomocytes
Source: PLoS One. 2022 May 6;17(5):e0267911. doi: 10.1371/journal.pone.0267911 (PMC9075664; doi:10.1371/journal.pone.0267911)
Supplement: S1 Fig — Eggs were injected with expression constructs according to [80] and evaluated at six days post fertilization as pluteus larvae. A-C. A larva injected with pONY_SpTrf-E2_GFP-X shows GFP expression in blastocoelar cells, in agreement with Ho et al. [77]. D-F. A larva injected with pONY_HE_GFP-X shows constitutive but mosaic GFP expression, in agreement with Solek et al. [75]. G-I. A larva injected with the empty vector, pONY_X_GFP-X, shows regions of auto-fluorescence, mostly in the gut. J-L. A non-injected larva shows regions of auto-fluorescence, mostly in the gut. Scale bars indicate 100 μm. (PDF) [file pone.0267911.s002.pdf]

Supporting Information

MA Barela Hudgell, LC Smith

Supporting Figure

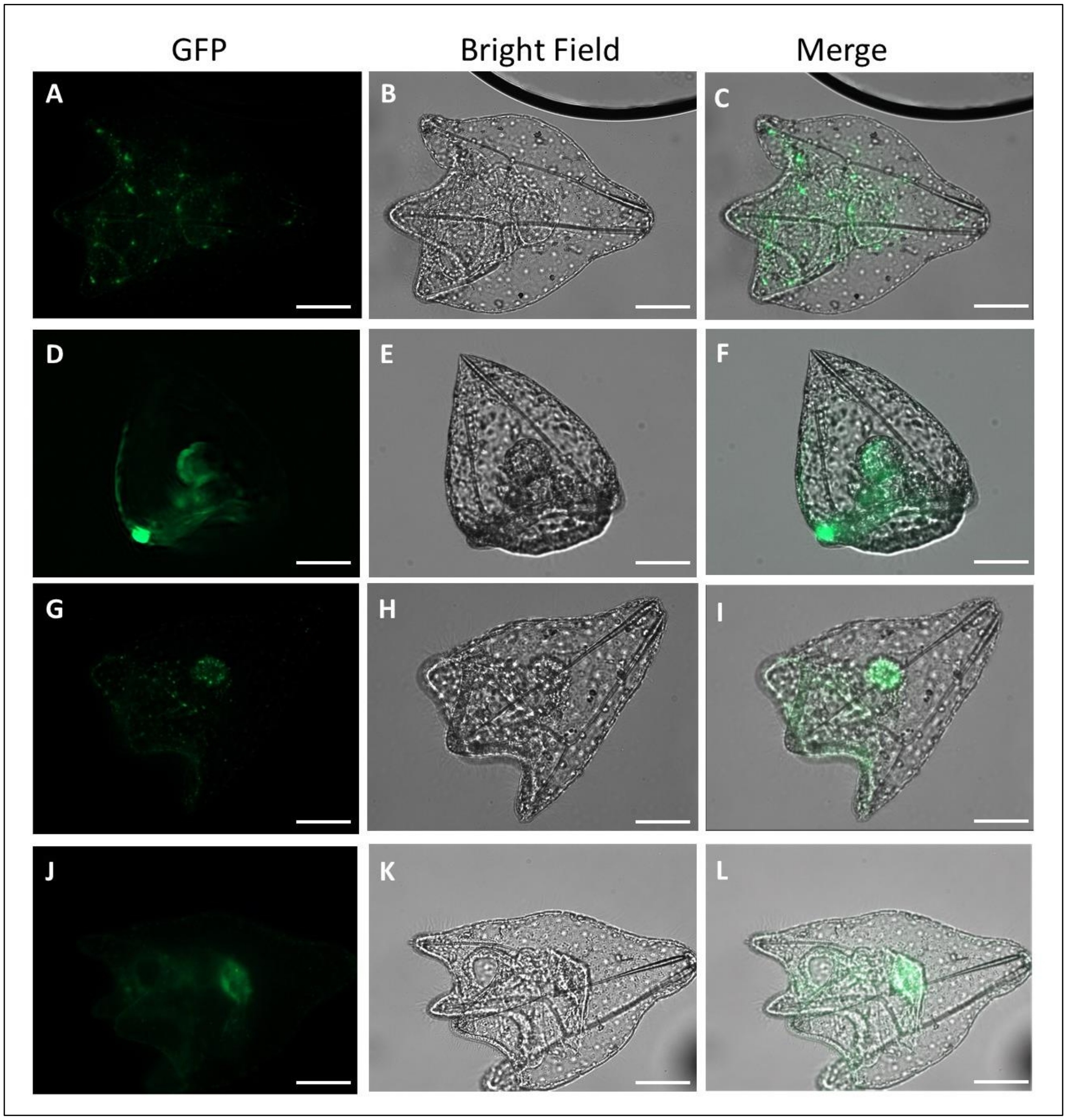

Fig S1. Pluteus larvae injected with *GFP* expression constructs show GFP expression. Eggs were injected with expression constructs according to (80) and evaluated at six days post fertilization as pluteus larvae. **A-C**. A larva injected with pONY_SpTrf-E2_GFP-X shows GFP expression in blastocoelar cells, in agreement with Ho et al. (77). **D-F**. A larva injected with pONY_HE_GFP-X shows constitutive but mosaic GFP expression, in agreement with Solek et al. (75). **G-I**. A larva injected with the empty vector, pONY_X_GFP-X, shows regions of auto-fluorescence, mostly in the gut. **J-L.** A non-injected larva shows regions of auto-fluorescence, mostly in the gut. Scale bars indicate 100 µm.
